# Influence of heart rate on right ventricular function assessed by right heart catheterization and echocardiography in healthy anesthetized dogs

**DOI:** 10.1186/s12917-022-03271-y

**Published:** 2022-05-06

**Authors:** Yunosuke Yuchi, Ryohei Suzuki, Haruka Kanno, Takahiro Saito, Takahiro Teshima, Hirotaka Matsumoto, Hidekazu Koyama

**Affiliations:** Laboratory of Veterinary Internal Medicine, School of Veterinary Medicine, Faculty of Veterinary Science, Nippon Veterinary and Life Science University, 1-7-1 Kyonan-cho, Musashino-shi, Tokyo, 180-8602 Japan

**Keywords:** Bowditch effect, Elastance, Force-frequency relation, Pressure–volume loop, Right ventricular contractility, Right ventricular performance, Strain, Strain rate, Two-dimensional speckle tracking echocardiography

## Abstract

**Background:**

Right ventricular (RV) functional assessment has received considerable attention in veterinary medicine since various diseases, such as cardiovascular, respiratory, endocrine, and neoplastic disease, may affect RV function. Heart rate (HR) is an important factor that can influence RV function through changes in loading condition and contractility. However, no study has yet evaluated the association between HR and RV function in the same individuals. This study aimed to evaluate the influence of elevated HR on RV function using right heart catheterization and echocardiography, and investigate the association between right heart catheterization and echocardiographic indices.

**Results:**

Right atrial pacing was performed in eight dogs at 120, 140, 160, and 180 bpm. With an increase in HR, the RV systolic volume, RV diastolic volume, and stroke volume significantly decreased; however, the cardiac output, end-systolic elastance (Ees), and effective arterial elastance (Ea) significantly increased. Significant changes were not observed in RV pressure and Ees/Ea. The RV area normalized by body weight, RV fractional area change normalized by body weight (RV FACn), and tricuspid annular plane systolic excursion normalized by body weight (TAPSEn) significantly decreased with increased HR. Peak systolic myocardial velocity of the lateral tricuspid annulus (RV s’), RV strain, and RV strain rate of only the RV free wall analysis (RV-SrL_3seg_) showed no significant changes with the increase in HR; however, there was an increase in the RV strain rate of the RV global analysis (RV-SrL_6seg_). Multiple regression analysis revealed that HR, RV FACn, and RV- SrL_6seg_ had significant associations with the Ees, and the TAPSEn and RV-SrL_3seg_ with Ees/Ea.

**Conclusions:**

Decreased venous return and shortened relaxation time decreased the RV FAC, TAPSE, RV s’, and RV strain, and might underestimate the RV function. Ees increased with the increase in HR, reflecting the myocardial force-frequency relation; as a result, RV-SrL_6seg_ could be a useful tool for Ees estimation. Additionally, the RV-SrL_3seg_ could detect RV performance, reflecting the balance between RV contractility and RV afterload.

## Background

Right ventricular (RV) function is affected by various diseases, such as cardiovascular, respiratory, endocrine, and neoplastic disease [1]. Recent studies have reported that pulmonary hypertension, which influences RV function through pressure overload, was a risk factor for poor outcomes in dogs with myxomatous mitral valve disease and respiratory disease/hypoxia [2, 3]. Additionally, RV dysfunction and dilatation were reported to have an association with shorter survival times in dogs with pulmonary hypertension [4]. Therefore, the morphological and functional assessment of the RV has received considerable attention in veterinary medicine, and right heart catheterization has been accepted as the gold standard diagnostic assessment. Catheterization-derived end-systolic elastance (Ees) and effective arterial elastance (Ea) indicate the intrinsic myocardial contractility and afterload, respectively [5]. Additionally, the ratio of Ees and Ea (Ees/Ea) could reflect the ventricular performance against afterload (i.e., ventricular-arterial coupling) [6–8]. However, this method is difficult to perform clinically in companion animals owing to the need for anesthesia. Therefore, echocardiography, an alternative non-invasive modality, is used most commonly by veterinarians to evaluate RV morphology and function. There are various RV functional indicators in veterinary medicine, including RV fractional area change (RV FAC), tricuspid annular plane systolic excursion (TAPSE), and tissue Doppler imaging-derived peak systolic myocardial velocity of the lateral tricuspid annulus (RV s’) [9–11]. Additionally, two-dimensional speckle tracking echocardiography (2D-STE) enables precise non-invasive evaluation of myocardial function [12]. However, high sensitivity to loading conditions could complicate echocardiographic evaluation of intrinsic RV function [13–16].

Heart rate (HR) is an important factor influencing cardiac loading conditions. Some echocardiographic indices for RV function have been shown to correlate with HR in previous clinical studies [9, 17]. Indeed, increased HR would reduce the preload (i.e., venous return) to the right heart [18], which could underestimate the RV function. However, increased HR activates cardiac contractility (force-frequency relation/Bowditch effect) [19]. Therefore, it is necessary to evaluate RV function while considering two factors: high sensitivity to loading conditions and force-frequency relation. To the authors’ knowledge, no study has yet evaluated the relationship between HR and RV function assessed by right heart catheterization and echocardiography in the same individual.

This study aimed to evaluate the influence of HR on RV morphology and function with RV pressure–volume loops and echocardiography in healthy anesthetized dogs. The echocardiographic variables which reflect RV function regardless of changes in HR were also explored. We hypothesized that increased HR could influence volume loading conditions and RV contractility, and would affect the echocardiographic RV variables; additionally, 2D-STE indices could reflect the precise changes in myocardial function.

## Results

All the datasets obtained from eight healthy anesthetized beagles were used for the statistical analyses. Right atrial pacing was completed in all the dogs; the range of HR at baseline was 70–112 bpm. The end-tidal partial pressure of carbon dioxide, tissue oxygen saturation, and oscillometric method-derived blood pressure were within the expected ranges for healthy dogs throughout the study.

### Hemodynamic measurements

**Fig. 1:**
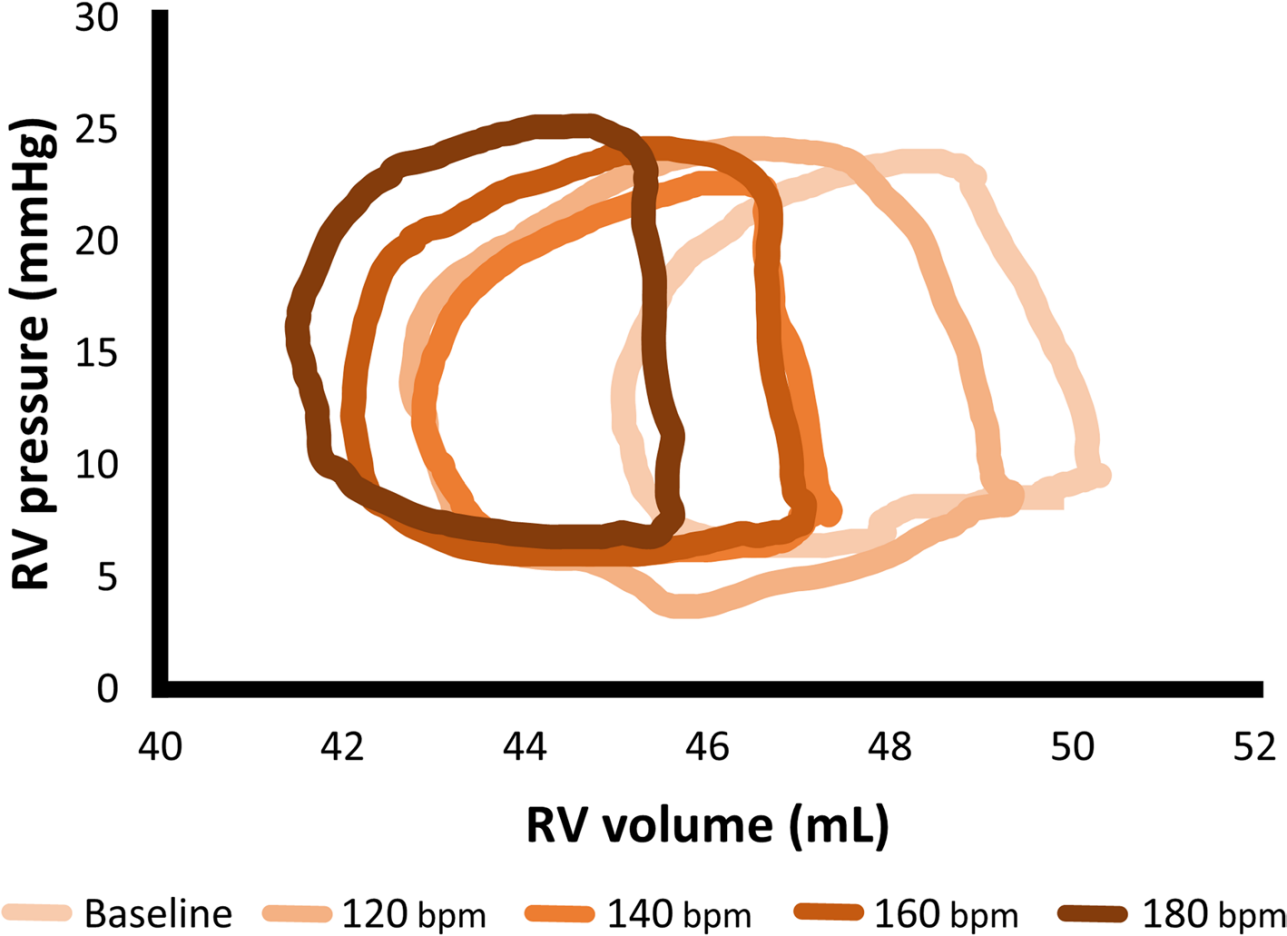
A representative data of right ventricular pressure–volume loops with the increased heart rate

The hemodynamic variable measurements obtained from the RV pressure–volume loops are summarized in Table 1. There were no significant changes in maximal and minimal RV pressure associated with the increase in HR; however, the maximal and minimal RV volume was significantly decreased (Fig. 1). Stroke volume was significantly decreased at 180 bpm compared with the baseline (*p* = 0.028). Contrarily, cardiac output was significantly increased at 140 bpm, 160 bpm, and 180 bpm compared with the baseline (*p* = 0.027, *p* = 0.027, and *p* = 0.048, respectively). The Ees and Ea were significantly increased with increased HR. The Ees/Ea showed no significant change with increased HR.

**Table 1:** Changes in hemodynamic variables at various right atrial pacing rates in 8 healthy anesthetized dogs

Variables	Baseline (range: 70 – 112)	120 bpm	140 bpm	160 bpm	180 bpm	p*
Maximal RV pressure (mmHg)	22.1 ± 2.7	22.6 ± 2.1	22.7 ± 1.9	22.5 ± 2.3	23.0 ± 2.4	0.453
Minimal RV pressure (mmHg)	6.6 ± 2.3	5.6 ± 1.9	5.7 ± 2.1	5.7 ± 2.0	5.9 ± 2.0	0.033
Maximal RV volume (mL)	55.8 ± 9.2	54.0 ± 8.8 ^a^	53.2 ± 9.3 ^a^	52.2 ± 8.7 ^a,b^	51.4 ± 8.6 ^a,b^	< 0.001
Minimal RV volume (mL)	48.6 ± 7.4	46.2 ± 7.5	45.6 ± 7.4	44.8 ± 6.9 ^a,b^	44.7 ± 6.8 ^a,b^	< 0.001
Stroke volume (mL)	7.2 ± 2.1	7.8 ± 2.2	7.6 ± 2.6	7.4 ± 2.5	6.7 ± 2.3 ^c^	0.026
Cardiac output (L/min)	0.72 ± 0.3	0.94 ± 0.3	1.1 ± 0.4 ^a^	1.2 ± 0.4 ^a^	1.2 ± 0.4 ^a^	< 0.001
Ees (mmHg/mL)	4.1 ± 0.9	4.6 ± 1.2	5.0 ± 1.5	4.9 ± 1.3 ^a^	5.1 ± 1.0 ^a^	0.018
Ea (mmHg/mL)	2.3 ± 1.0	2.1 ± 0.6	2.2 ± 0.9	2.2 ± 0.7	2.6 ± 0.9^b^	0.047
Ees/Ea	2.0 ± 0.5	2.3 ± 0.6	2.4 ± 0.6	2.3 ± 0.4	2.1 ± 0.5	0.313

Data are represented as mean ± standard deviation for normally distributed data or median (interquartile range) for non-normally distributed data

*Ea* effective arterial elastance, *Ees* end-systolic elastance, *RV* right ventricular

^*^P value of the repeated-measures analysis of variance

^a^The value is significantly different from baseline (*p* < 0.050)

^b^The value is significantly different from 120 bpm (*p* < 0.050)

^c^The value is significantly different from 160 bpm (*p* < 0.050)

### Echocardiographic indices for the right heart

Table 2 shows the results of the echocardiographic indices for RV morphology and function. Right atrial pacing at 160 and 180 bpm significantly decreased the end-diastolic and end-systolic RV area normalized by body weight (RVEDA index and RVESA index, respectively). Additionally, the RV fractional area change normalized by body weight (RV FACn) and the tricuspid annular plane systolic excursion normalized by body weight (TAPSEn) significantly decreased with increased HR. The tissue Doppler imaging-derived RV s’ showed no significant change with increased HR.

**Table 2:** Changes in echocardiographic variables at various right atrial pacing rates in 8 healthy anesthetized dogs

Variables	Baseline (range: 70 - 112)	120 bpm	140 bpm	160 bpm	180 bpm	p*
RVEDA index (cm^2^/kg^0.624^)	1.21 (1.09 - 1.30)	1.16 (1.05 - 1.25)	1.10 (0.95 - 1.21)	0.97^a,b^ (0.96 - 1.03)	0.88^a,b,c^ (0.76 - 0.96)	< 0.001
RVESA index (cm^2^/kg^0.628^)	0.71 ± 0.1	0.67 ± 0.1	0.64 ± 0.1 ^a^	0.60 ± 0.1^a^	0.55 ± 0.1^a,b,c^	< 0.001
RV FACn (%/kg^−0.097^)	51.8 ± 3.9	51.2 ± 5.9	51.3 ± 6.0	46.6 ± 4.5	46.4 ± 2.9^a^	< 0.001
TAPSEn (mm/kg^0.284^)	4.4 ± 0.6	4.1 ± 0.7	3.6 ± 0.6^a,b^	3.3 ± 0.5^a,b,c^	2.8 ± 0.5^a,b,c,d^	< 0.001
RV s’ (cm/s)	6.6 ± 1.0	7.1 ± 1.6	6.9 ± 1.7	6.9 ± 1.6	6.7 ± 1.4	0.345

Data are represented as mean ± standard deviation for normally distributed data or median (interquartile range) for non-normally distributed data

*RV FACn* right ventricular fractional area change normalized by body weight, *RV s’* peak systolic myocardial velocity of the lateral tricuspid annulus, *RVEDA index* end-diastolic right ventricular area normalized by body weight, *RVESA index* end-systolic right ventricular area normalized by body weight, *TAPSEn* tricuspid annular plane systolic excursion normalized by body weight

^*^P value of the repeated-measures analysis of variance or the Friedman test

^a^The value is significantly different from baseline (*p* < 0.050)

^b^The value is significantly different from 120 bpm (*p* < 0.050)

^c^The value is significantly different from 140 bpm (*p* < 0.050)

^d^The value is significantly different from 160 bpm (*p* < 0.050)

**Fig. 2:**
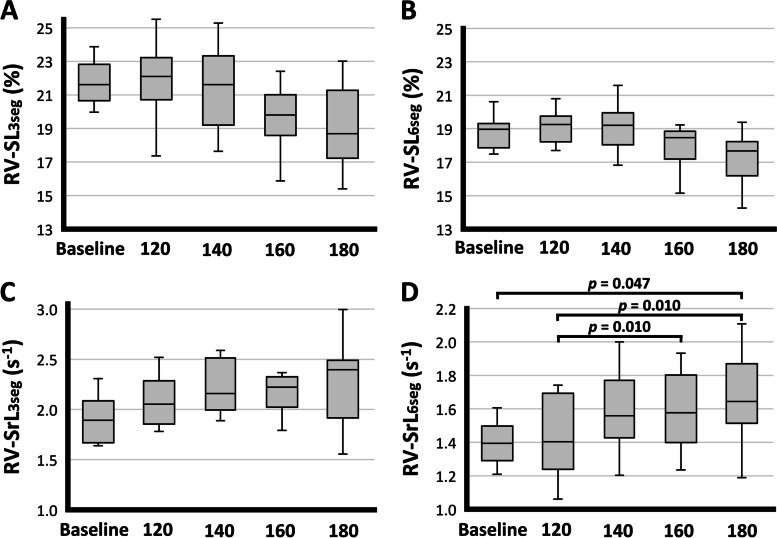
Box and Whisker plots of right ventricular strain and strain rate in 8 healthy dogs. **A**: right ventricular (RV) strain of only RV free wall analysis (RV-SL_3seg_). **B**: RV strain of RV global analysis (RV-SL_6seg_). **C**: RV strain rate of only RV free wall analysis (RV-SrL_3seg_). **D**: RV strain rate of RV global analysis (RV-SrL_6seg_). The Whiskers indicate the range of values obtained, the box extends from the 25th to the 75th percentile, and the horizontal bar in the box represents the median

Regarding the 2D-STE indices, all the myocardial segments were included in the statistical analyses. At all pacing rates, the mean ± standard deviation of frame rates of the echocardiographic cineloops used for the 2D-STE analyses were 131 ± 4 frames per second. The RV longitudinal strain (RV-SL) of each segmental analysis showed no significant change with increased HR, although RV-SL had the tendency to decrease at 160 bpm and 180 bpm (Fig. 2A, 2B). The RV strain rate of only RV free wall analysis (RV-SrL_3seg_) showed no significant change with the increased HR (*p* = 0.090) (Fig. 2C). However, the RV-SrL of the RV global analysis (RV-SrL_6seg_) was significantly increased with increased HR (Fig. 2D).

### Single and multiple regression analyses

Table 3 shows the Pearson’s and Spearman’s correlation coefficients between HR and hemodynamic and echocardiographic variables evaluated in this study. For the RV pressure–volume loops-derived variables, the maximal and minimal RV volumes had significant negative correlations with HR, whereas cardiac output and Ees had significant positive correlations with HR. The RV pressure, stroke volume, and Ea showed no significant correlations with HR. For the echocardiographic variables, RVEDA index, RVESA index, RV FACn, TAPSEn, RV-SL_3seg_, and RV-SL_6seg_ had significant negative correlations with HR. In contrast, RV-SrL_3seg_ and RV-SrL_6seg_ showed significant positive correlations with HR.

**Table 3:** Correlation coefficients between HR and hemodynamic and echocardiographic variables

Variables	Heart rate	
	Correlation coefficients	p*
Maximal RV pressure (mmHg)	0.08	0.620
Minimal RV pressure (mmHg)	-0.07	0.647
Maximal RV volume (mL)	-0.20	0.021
Minimal RV volume (mL)	-0.20	0.021
Stroke volume (mL)	-0.07	0.677
Cardiac output (L/min)	0.47	0.002
Ees (mmHg/mL)	0.25	0.011
Ea (mmHg/mL)	0.12	0.450
Ees/Ea	0.06	0.729
RVEDA index (cm^2^/kg^0.624^)	-0.68	< 0.001
RVESA index (cm^2^/kg^0.628^)	-0.56	< 0.001
RV FACn (%/kg^−0.097^)	-0.40	0.010
TAPSEn (mm/kg^0.284^)	-0.72	< 0.001
RV s’ (cm/s)	0.04	0.828
RV-SL_3seg_ (%)	-0.37	0.017
RV-SrL_3seg_ (s^−1^)	0.42	0.008
RV-SL_6seg_ (%)	-0.31	0.054
RV-SrL_6seg_ (s^−1^)	0.44	0.005

*3seg* RV free wall analysis, *6seg* RV global analysis, *Ea* effective arterial elastance, *Ees* end-systolic elastance, *RV* right ventricular, *RV* FACn: RV fractional area change normalized by body weight, *RV s’* peak systolic myocardial velocity of the lateral tricuspid annulus, *RVEDA index* end-diastolic RV area normalized by body weight, *RVESA index* end-systolic RV area normalized by body weight, *RV-SL* RV strain, *RV-SrL* systolic RV strain rate, *TAPSEn* tricuspid annular plane systolic excursion normalized by body weight

^*^P value of the Pearson’s or Spearman’s correlation coefficient

**Fig. 3:**
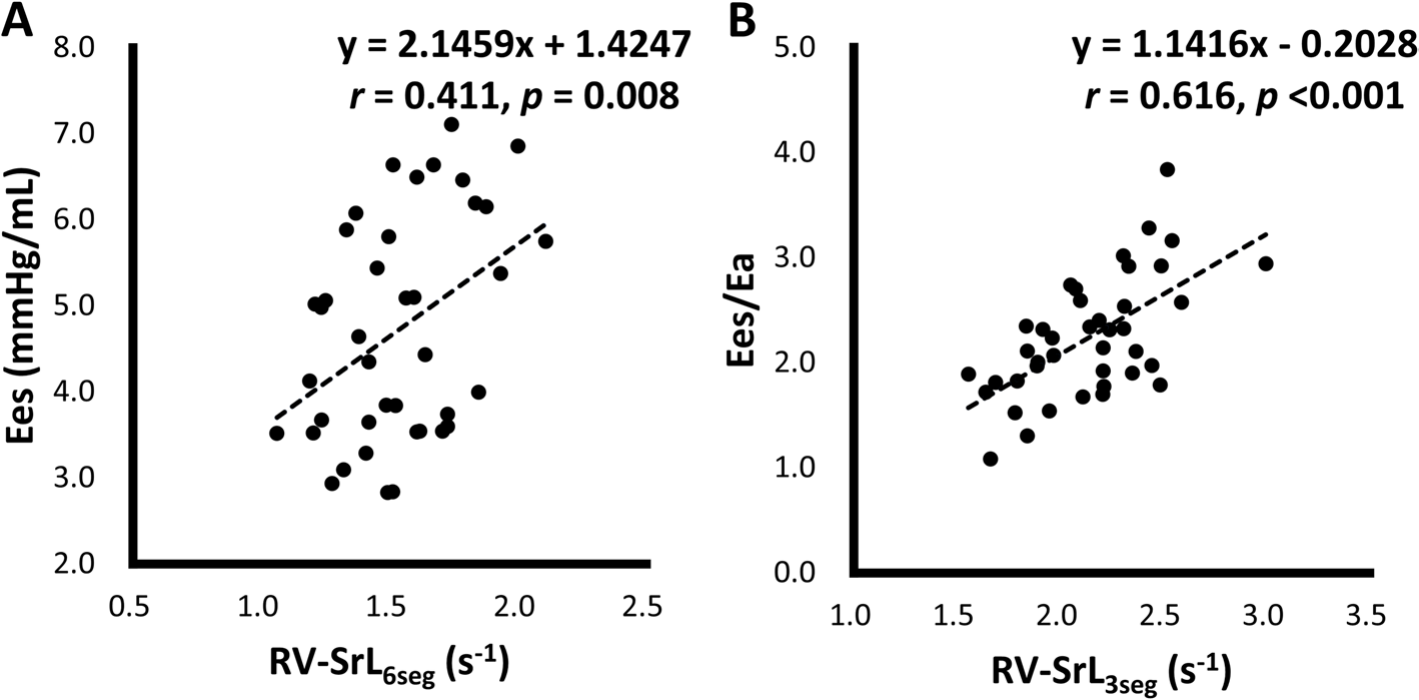
Correlations between hemodynamic indices and right ventricular strain rate. **A**: Correlation between end-systolic elastance (Ees) and right ventricular (RV) strain rate of RV global analysis (RV-SrL_6seg_). **B**: Correlation between Ees to effective arterial elastance ratio (Ees/Ea) and RV strain rate of only RV free wall analysis (RV-SrL_3seg_)

Table 4 summarizes the results of single regression analyses to evaluate the association between hemodynamic variables (Ees and Ees/Ea) and echocardiographic indices. HR, RV FACn, and RV-SrL_6seg_ showed significant associations with Ees in the single linear regression analysis, and the highest moderate correlation with Ees was observed in the RV-SrL_6seg_ (*r* = 0.41) (Fig. 3A). Furthermore, the RV FACn, TAPSEn, RV s’, RV-SL_3seg_, RV-SL_6seg_, RV-SrL_3seg_, and RV-SrL_6seg_ showed significant associations with Ees/Ea in the single linear regression analysis; the RV-SrL_3seg_ had the highest correlation with Ees/Ea (*r* = 0.62) (Fig. 3B). After adjusting for the confounding factors with multiple regression analysis, the HR, RV FACn, and RV-SrL_6seg_ were significantly associated with Ees (*p* < 0.001, multiple adjusted *r*^*2*^ = 0.44), while TAPSEn and RV-SrL_3seg_ were significantly associated with Ees/Ea (*p* < 0.001, multiple adjusted *r*^*2*^ = 0.46).

**Table 4:** Results of single regression analyses to evaluate the association between hemodynamic and echocardiographic variables

Variables	Ees		Ees/Ea	
	regression coefficient (95% CI)	*p*	regression coefficient (95% CI)	*p*
HR (bpm)	0.01 (0.00 – 0.02)	0.039	0.00 (0.00 – 0.01)	0.642
RVEDA index (cm^2^/kg^0.624^)	-1.79 (-4.03 – 0.45)	0.114	0.19 (-0.85 – 1.23)	0.713
RVESA index (cm^2^/kg^0.628^)	-0.80 (-4.69 – 3.09)	0.680	-0.68 (-2.41 – 1.05)	0.431
RV FACn (%/kg^−0.097^)	-0.09 (-0.17 – -0.02)	0.010	0.05 (0.01 – 0.08)	0.006
TAPSEn (mm/kg^0.284^)	-0.34 (-0.84 – 0.16)	0.176	0.20 (-0.02 – 0.42)	0.070
RV s’ (cm/s)	-0.07 (-0.34 – 0.21)	0.630	0.21 (0.11 – 0.32)	< 0.001
RV-SL_3seg_ (%)	-0.06 (-0.20 – 0.09)	0.414	0.10 (0.04 – 0.15)	0.002
RV-SrL_3seg_ (s^−1^)	0.60 (-0.74 – 1.94)	0.374	1.14 (0.66 – 1.62)	< 0.001
RV-SL_6seg_ (%)	0.03 (-0.14 – 0.20)	0.745	0.08 (0.01 – 0.15)	0.033
RV-SrL_6seg_ (s^−1^)	2.15 (0.58 – 3.71)	0.008	1.08 (0.39 – 1.76)	0.003

*3seg* right ventricular free wall analysis, *6seg* right ventricular global analysis, *HR* heart rate, *RV FACn* right ventricular fractional area change normalized by body weight, *RV s’* peak systolic myocardial velocity of the lateral tricuspid annulus, *RVEDA index* end-diastolic right ventricular area normalized by body weight, *RVESA index* end-systolic right ventricular area normalized by body weight, *RV-SL* right ventricular strain, *RV-SrL* systolic right ventricular strain rate, *TAPSEn* tricuspid annular plane systolic excursion normalized by body weight

### Intra- and interobserver measurement variability

Results of the intra- and inter-observer measurement variability are summarized in Table 5. All echocardiographic indices had low intra-observer measurement variability [20]. Additionally, all echocardiographic indices met the definition of low inter-observer measurement variability [20].

**Table 5:** Intra- and inter-observer measurement variability of the echocardiographic variables

Variables	Intra-observer		Inter-observer	
	CV	ICC	CV	ICC
RVEDA index	2.2	0.97*	6.1	0.91*
RVESA index	4.4	0.95*	7.4	0.83*
RV FACn	5.0	0.91*	9.1	0.85*
TAPSEn	1.8	0.99*	3.5	0.99*
RV s'	1.3	0.99*	2.2	0.99*
RV-SL_3seg_	4.1	0.93*	7.2	0.88*
RV-SrL_3seg_	5.9	0.89*	9.5	0.91*
RV-SL_6seg_	5.2	0.98*	7.6	0.96*
RV-SrL_6seg_	5.7	0.93*	6.9	0.87*

*3seg* right ventricular free wall analysis, *6seg* right ventricular global analysis, *CV* coefficient of variation, *ICC* inter- or intra-class correlation coefficients, *RV FACn* right ventricular fractional area change normalized by body weight, *RV s'* peak systolic myocardial velocity of the lateral tricuspid annulus, *RVEDA index* end-diastolic right ventricular area normalized by body weight, *RVESA index* end-systolic right ventricular area normalized by body weight, RV-SL right ventricular strain, *RV-SrL* systolic right ventricular strain rate, *TAPSEn* tricuspid annular plane systolic excursion normalized by body weight

^*^ Within a row, ICC values were considered significant (*p* < 0.050)

## Discussion

In this study, the RV size indicators, including catheterization-derived RV volume and echocardiography-derived RVEDA and RVESA, significantly decreased with increased HR. These results might reflect the incomplete myocardial relaxation and decreased venous return caused by higher HR. As the HR increased, certain echocardiographic indices, including RV FACn, TAPSEn, RV s’, and RV-SL decreased. However, catheterization-derived Ees increased, suggesting that these echocardiographic indices were sensitive to the decrease in preload and thus could underestimate RV systolic function. Nonetheless, the RV-SrL_6seg_ showed a significant association with the Ees, as did the RV-SrL_3seg_ with the Ees/Ea. Our results indicated that the 2D-STE-derived RV-SrL could reflect intrinsic RV contractility and RV performance regardless of fluctuations in HR.

Several conventional echocardiographic indices used for the assessment of RV function, including RV FACn and TAPSEn, significantly decreased with increased HR; however, the Ees, which indicates intrinsic cardiac contractility, increased. Additionally, the 2D-STE-derived RV-SL and RV s’ showed no significant increases with increased HR; interestingly, RV-SL tended to decrease at rates of 160 and 180 bpm. An increase in HR would shorten ejection time and relaxation time, and decrease the venous return (incomplete relaxation [19]). A previous experimental study has reported that acute volume overload increased echocardiographic indices such as TAPSE, RV FAC, RV s’, and the 2D-STE derived RV-SL and RV-SrL [16]. Our results also suggested that shortened ejection and relaxation times and a decreased volume loading condition could underestimate RV systolic function when using conventional echocardiographic indices and 2D-STE-derived RV-SL.

The RV-SrL showed a significant positive correlation with HR as well as Ees, although certain conventional echocardiographic indices, such as RV FACn, TAPSEn, and RV s’, showed either no correlations or significant negative correlations. The 2D-STE method enables assessment of the RV myocardial function without the influences of angle-dependency, translation, or tethering of the heart [12, 21], and supports the intrinsic myocardial function evaluation of the whole RV. Our results may suggest that the 2D-STE-derived RV functional indices could precisely evaluate RV function. Specifically, RV-SrL_6seg_ had a significant association with Ees, but RV-SL and RV-SrL_3seg_ did not. A previous study reported that the left ventricular strain rate more closely reflects myocardial contractility with load-independency than did left ventricular strain [22]. Additionally, several experimental studies have reported that the interventricular septum plays an important role in RV cardiac output [23, 24]. Therefore, our results indicated that the myocardial motility of the global RV, including not only the RV free wall but also the interventricular septum, might contribute to RV contractility; additionally, the RV-SrL_6seg_ might reflect intrinsic RV myocardial contractility regardless of HR. However, the 2D-STE method could not separate the myocardial function of the interventricular septum into right and left ventricular components. Further studies that also assess left ventricular function are warranted in the future.

In addition to the increase in Ees associated with the force-frequency relation, Ea was significantly increased at 180 bpm when compared with that at 120 bpm. Previous canine studies reported that the left ventricular Ea approximates peripheral vascular resistance multiplied by HR [25, 26]. Although there was no significant correlation between HR and Ea, our results indicated that an excessively increased HR might increase the Ea of the right and left ventricles. With the increase in both Ees and Ea, the Ees/Ea was stable even when HR increased. In this study, the RV-SrL_3seg_ and TAPSEn demonstrated an association with the Ees/Ea in the regression analyses, and the RV-SrL_3seg_ had the highest correlation with Ees/Ea. The Ees/Ea is an indicator of RV performance, reflecting the relationship between RV contractility and afterload [6–8]. Of the indices evaluated in this study, our results suggest that the RV-SrL_3seg_ may be the most suitable tool to evaluate RV performance regardless of HR fluctuation. Additionally, RV-SrL_3seg_ had a higher correlation with Ees/Ea than with TAPSEn in this study. Since RV myocardial motility could vary in different regions of the RV free wall [27, 28], TAPSE (which evaluates only basal regional RV function) would not have sufficient power to reflect the RV performance. Evaluation of the entire RV free wall (RV-SrL_3seg_) may be necessary to estimate the RV performance.

This study had certain limitations. The primary limitation of this study was the relatively small sample size. As we did not perform an a priori power calculation, the small sample size could have influenced the statistical power necessary to detect the changes in specific RV morphological and functional variables as HR increased. Second, the influence of HR on RV morphology and function was assessed using healthy anesthetized beagles. Certain drugs used for anesthesia, especially isoflurane, could have affected RV afterload and contractility [29, 30]. Additionally, our results could differ in dogs of other breeds or those with cardiac diseases affecting RV function. Third, the frame rates of echocardiography cineloops used for the 2D-STE analysis were constant throughout the study. The relatively decreased number of frames at higher HRs could affect the results of the 2D-STE indices. However, all 2D-STE indices were obtained from the echocardiographic cineloops with sufficient frame rates [31, 32]. Third, this study did not measure hemodynamic variables other than RV pressure–volume loops. Changes might have occurred in pulmonary artery pressure and right atrial pressure during anesthesia and/or pacing that might have influenced our results. Finally, we evaluated only the RV longitudinal myocardial function. The RV circumferential function could also contribute to RV performance [33, 34].

## Conclusions

In this study, echocardiographic indices of RV function, namely RV FACn, TAPSEn, RV s’, and 2S-STE-derived RV-SL, decreased with increased HR. This indicated that the aforementioned indices were sensitive to decreased venous return and incomplete relaxation and could underestimate RV systolic function. However, the intrinsic RV contractility assessed with Ees was increased according to the force-frequency relation/Bowditch effect; RV-SrL_6seg_ could be a useful tool to estimate the Ees. Additionally, the RV-SrL_3seg_ may detect RV performance assessed by Ees/Ea, reflecting the balance between RV contractility and afterload.

## Methods

### Study design and setting

This was a hypothesis-driven, experimental study. All procedures followed the Guidelines for Institutional Laboratory Animal Care and Use of Nippon Veterinary and Life Science University in Tokyo, Japan. The study was approved by the ethical committee for laboratory animal use of the Nippon Veterinary and Life Science University in Tokyo, Japan (approval No. 2020S-46).

### Animals

Eight healthy beagles owned by our laboratory (male/female: 4/4; age [mean ± standard deviation]: 1.4 ± 0.1 years; body weight [mean ± standard deviation]: 9.9 ± 1.0 kg) were used in this study. Each dog was determined to be healthy based on the findings of a complete physical examination, complete blood cell count (poch-100*i*V Diff, Sysmex Corporation, Hyogo, Japan), serum blood chemistry profile (FDC7000V, FUJIFILM Corporation, Kanagawa, Japan), electrocardiography (D320, FUKUDA M-E KOGYO Co, LTD, Tokyo, Japan), thoracic and abdominal radiography (FCR PRIMA V and FD0078-V Station T, FUJIFILM Corporation, Kanagawa, Japan), transthoracic and abdominal ultrasonography (Vivid iq, GE Healthcare, Tokyo, Japan), and oscillometric blood pressure measurement (BP100DII, FUKUDA M-E KOGYO Co, LTD, Tokyo, Japan).

### Study protocol

All dogs were premedicated with butorphanol tartrate (0.2 mg/kg, IV) and midazolam hydrochloride (0.2 mg/kg, IV), and received cefazolin sodium hydrate (20.0 mg/kg, IV). Anesthesia was induced with propofol (4.0 to 6.0 mg/kg, IV to effect) and dogs were intubated and maintained on 1.5% isoflurane. Each dog received an intravenous infusion of lactated Ringer’s solution at a rate of 3.0 mL/kg/h throughout the experiments. Pressure-controlled mechanical ventilation at a rate of 10 breaths per minute was maintained throughout the study. End-tidal partial pressure of carbon dioxide, transcutaneously measured tissue oxygen saturation, heart rate, and oscillometric blood pressure were assessed throughout the study with a multiparameter monitor (AM130, FUKUDA M-E KOGYO Co, LTD, Tokyo, Japan).

All dogs were restrained in a supine position, and the left and right sides of the neck were clipped, aseptically prepared, and draped. A 6-Fr sheath introducer (Radifocus Introducer IIH, Terumo Corporation, Tokyo, Japan) was inserted into the left and right jugular veins using a Seldinger retainment technique. After the insertion of the sheath introducers, the dogs were positioned in left lateral recumbency. A 5-Fr pressure–volume catheter (Ventri-Cath 507, Millar Inc, Texas, US) was positioned in the right ventricle with the aid of fluoroscopic guidance (Brivo OEC 785, GE Healthcare Japan, Tokyo, Japan) and manually adjusted to obtain accurate RV pressure–volume loops. Additionally, a 4-Fr pacing catheter (TL-410, Abbott Medical Japan LLC, Tokyo, Japan) was positioned in the right atrium and manually adjusted to obtain accurate right atrial pacing with the aid of fluoroscopic guidance and electrocardiography. After a 10-min stabilization period, hemodynamic measurements (VPR-1003, Unique Medical Co, LTD, Tokyo, Japan) and echocardiography (Vivid iq, GE Healthcare, Tokyo, Japan) were performed to obtain baseline data. Right atrial pacing was subsequently performed using an external programmable pacemaker (Model SEP-101, Star Medical Inc, Tokyo, Japan); heart rates of 120 bpm, 140 bpm, 160 bpm, and 180 bpm were randomly generated. After 5 min of continuous right atrial pacing, hemodynamic measurements and echocardiography were performed at each pacing rate. When all of the experimental protocols were completed, the catheters and sheath introducers were removed and manual astriction was performed at the catheterization sites. All dogs were then recovered from anesthesia, and administered cephalexin (20.0 mg/kg, PO, BID for 3 days) and robenacoxib (2.0 mg/kg, SC, SID as needed). After completing the study protocol, all dogs were transferred for another study at our institution.

### Right heart catheterization

Right heart catheterizations and hemodynamic analyses were performed by a single observer using a ventricular pressure–volume measurement device (VPR-1003, Unique Medical Co, LTD, Tokyo, Japan) and analysis software (Integral3, Unique Medical Co, LTD, Tokyo, Japan). RV pressure–volume loops were recorded for 30 s.

The mean values of five consecutive pressure–volume loops at the end of the expiratory phase were used in the statistical analysis to eliminate the influence of respiratory variations [35]. The following variables were measured as RV hemodynamic indicators: maximal and minimal RV pressure, maximal and minimal RV volume, stroke volume, cardiac output, Ees, Ea, and Ees/Ea. Stroke volume was calculated as the difference in maximal and minimal RV volume, and cardiac output was defined as stroke volume multiplied by HR. The Ees was calculated using the RV single-beat method as previously described [36].

### Echocardiographic evaluation of the right heart

Conventional 2D, M-mode, and Doppler examinations were performed using an echocardiography system (Vivid iq, GE Healthcare, Tokyo, Japan) and a 3.5–6.9 MHz transducer. Lead II electrocardiography was performed simultaneously and results were displayed on the images. Data were obtained from at least 5 consecutive cardiac cycles in each dog while in lateral recumbency. All images were analyzed using an offline workstation (EchoPAC PC, Version 204, GE Healthcare, Tokyo, Japan).

The means of the five consecutive cardiac cycles measured during paced rhythms were used in all analyses of the right heart echocardiographic indices. Furthermore, all indices were measured at the end-expiratory phase. The RVEDA and RVESA were measured as RV morphological indicators. These indices were obtained from the left apical four-chamber view optimized for the right heart (RV focus view) [10, 37, 38], and were measured by tracing the endocardial border of the RV inflow region at end-diastole and end-systole, excluding the papillary muscles [11, 37]. To eliminate the effect of different body sizes, the RVEDA and RVESA were normalized using the following formulae [38]:

$$\mathrm{RVEDA}\;\mathrm{index}\:=\:(\mathrm{RVEDA}\;\lbrack\mathrm{cm}^2\rbrack)/{(\mathrm{body}\;\mathrm{weight}\;\lbrack\mathrm{kg}\rbrack)}^{0.624}$$

$$\mathrm{RVESA}\;\mathrm{index}\:=\:(\mathrm{RVESA}\;\lbrack\mathrm{cm}^2\rbrack)/{(\mathrm{body}\;\mathrm{weight}\;\lbrack\mathrm{kg}\rbrack)}^{0.628}$$

For assessing RV function, the following indices were measured: TAPSE, RV FAC, and RV s’. These indices were obtained from the RV focus view [10, 37, 38]. The TAPSE was measured using the B-mode cineloops as the total displacement of the tricuspid annulus from end-diastole to end-systole [39, 40]. The RV FAC was calculated using the RVEDA and RVESA [37]:

$$\mathrm{RV}\;\mathrm{FAC}\;(\%)\:=\:(\lbrack\mathrm{RVEDA}\textemdash\mathrm{RVESA}\rbrack/\mathrm{RVEDA})\:\times\:100$$

The TAPSE and RV FAC values normalized by body weight (TAPSEn and RV FACn, respectively) were calculated using the following formulae [37, 41]:

$$\mathrm{TAPSEn}\:=\:(\mathrm{TAPSE})/{(\mathrm{body}\;\mathrm{weight}\;\lbrack\mathrm{kg}\rbrack)}^{0.33}$$

$$\mathrm{RV}\;\mathrm{FACn}\:=\:(\mathrm{RV}\;\mathrm{FAC})/{(\mathrm{body}\;\mathrm{weight}\;\lbrack\mathrm{kg}\rbrack)}^{-0.097}$$

The RV s’ was measured as the peak systolic velocity obtained from the tissue Doppler imaging-derived lateral tricuspid annular motion wave [37].

#### 2D-STE

**Fig. 4:**
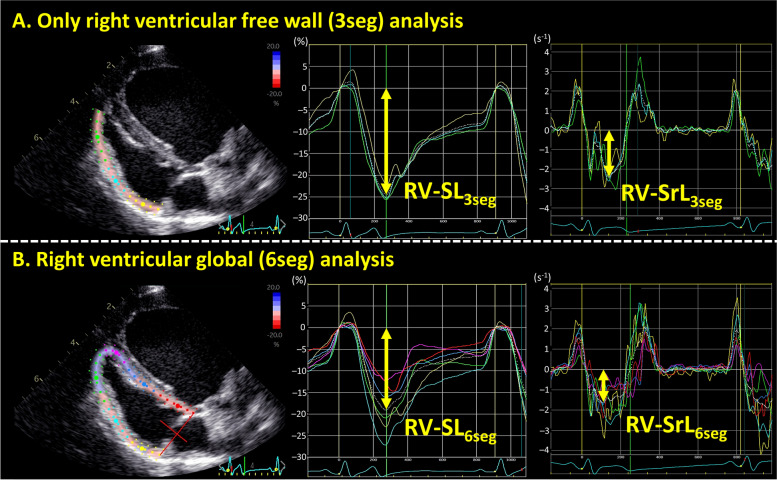
Right ventricular strain and strain rate obtained by two-dimensional speckle tracking echocardiography. **A**: Right ventricular strain and strain rate of only RV free wall analysis (RV-SL_3seg_ and RV-SrL_3seg_). **B**: Right ventricular strain and strain rate of RV global analysis (RV-SL_6seg_ and RV-SrL_6seg_)

All 2D-STE analyses were performed by the same investigator using the same ultrasound and offline workstation as those for standard echocardiography. The strain and strain rate were obtained from the RV focus view using the left ventricular four-chamber algorithms [39]. The region of interest for the 2D-STE was defined by manually tracing the RV endocardial border. The RV free wall analysis (3seg) was performed by tracing from the level of the lateral tricuspid annulus to the RV apex for the longitudinal strain (RV-SL_3seg_), as well as the systolic strain rate (RV-SrL_3seg_) (Fig. 4A). The RV global analysis (6seg) was also performed by tracing from the lateral tricuspid annulus to the septal tricuspid annulus (including the interventricular septum) via the RV apex for the longitudinal strain (RV-SL_6seg_), as well as the systolic strain rate (RV-SrL_6seg_) (Fig. 4B). Manual adjustments were performed to include and track the entire myocardial thickness throughout the cardiac cycle, if necessary. When the automated software could not track the myocardial regions, the regions of interest were retraced and recalculated. The RV-SL was defined as the absolute value of the negative peak value obtained from the strain wave. The RV-SrL was defined as the absolute value of the negative peak value obtained from the strain rate wave during systole [42].

### Intra- and inter-observer measurement variability

Intra-observer measurement variability was performed by the same observer who performed all the echocardiographic analyses. The echocardiographic indices of RV morphology and function for three dogs at baseline and at each pacing rate were measured on different days using the same echocardiogram and heart cycles. A second blinded observer measured the same echocardiographic indices to obtain interobserver measurement variability using the same five cardiac cycles. The mean values from two different days and from two different observers assessing the same cardiac cycles were used to evaluate intra- and interobserver measurement variability, respectively.

### Statistical analysis

All statistical analyses were performed using R software version 2.8.1. Continuous variables were reported as mean ± standard deviation for normally distributed data and median (interquartile range) for non-normally distributed data.

Data normality was tested using the Shapiro–Wilk test. Continuous variables were compared between the baseline and each pacing rate using a repeated-measures analysis of variance, with subsequent pairwise comparisons using Tukey’s multiple comparison test for normally distributed data. The Friedman test with subsequent pairwise comparisons using the Steel–Dwass test was used for non-normally distributed data. To evaluate the influence of HR on RV morphological and functional variables, Pearson’s and Spearman’s correlation coefficients were calculated. Additionally, univariate and multiple regression analyses were performed to identify the relationships between specific RV hemodynamic indices (Ees and Ees/Ea) and echocardiographic indices. The correlation was considered to be strong if the absolute value of the correlation coefficient |*r*| was > 0.7, moderate if 0.4 to 0.7, weak if 0.2 to 0.4, and no correlation if < 0.2. The indices with *p* < 0.10 in the univariate analysis were entered into the multiple regression analysis.

Intra- and inter-observer measurement variability was quantified by the coefficient of variation (CV) using a root mean square method, as previously described [43]. Additionally, Intra- and inter-class correlation coefficients (ICC) were also used to evaluate measurement variability. Low measurement variability was defined as CV < 10.0 and ICC > 0.7. Statistical significance was set at *p* < 0.05.

## Data Availability

The datasets used and/or analyzed during the current study are available from the corresponding author upon reasonable request.

## Abbreviations

2D-STE: Two-Dimensional Speckle Tracking Echocardiography
3seg: Right Ventricular Free Wall Analysis
6seg: Right Ventricular Global Analysis
CV: Coefficient of Variation
HR: Heart Rate
ICC: Inter- or Intra-Class Correlation Coefficients
Ea: Effective Arterial Elastance
Ees: End-systolic EChanges in hemodynamic variables at various right atrial pacing ratlastance
RV: Right Ventricle/Right Ventricular
RV FACn: Right Ventricular Fractional Area Change Normalized by Body Weight
RVEDA index: End-diastolic Right Ventricular Area Normalized by Body Weight
RVESA index: End-systolic Right Ventricular Area Normalized by Body Weight
RV s’: Peak Systolic Myocardial Velocity of the Lateral Tricuspid Annulus
RV-SL: Right Ventricular Longitudinal Strain
RV-SrL: Systolic Right Ventricular Strain Rate
TAPSEn: Tricuspid Annular Plane Systolic Excursion Normalized by Body Weight
